# Supplementary material for: The Development of Prenatal Muscle Satellite Cells (MuSCs) and Their Epigenetic Modifications During Skeletal Muscle Development in Yak Fetus
Source: Biology (Basel). 2024 Dec 23;13(12):1091. doi: 10.3390/biology13121091 (PMC11673279; doi:10.3390/biology13121091)
Supplement: Supplementary file 1 [file biology-13-01091-s001.zip › biology-3323722-supplementary.pdf]

a

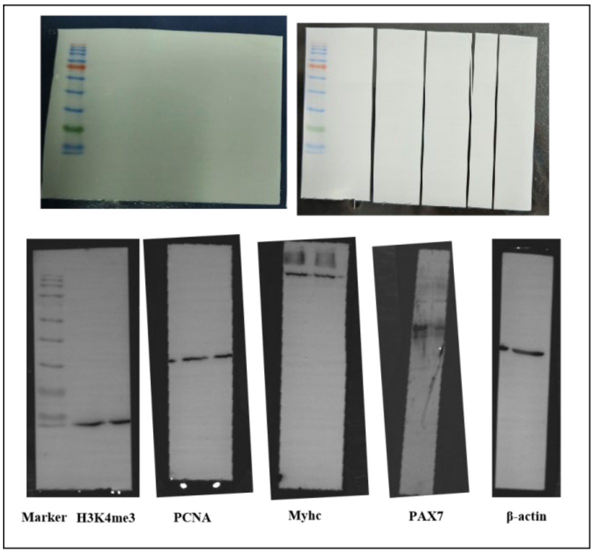

b

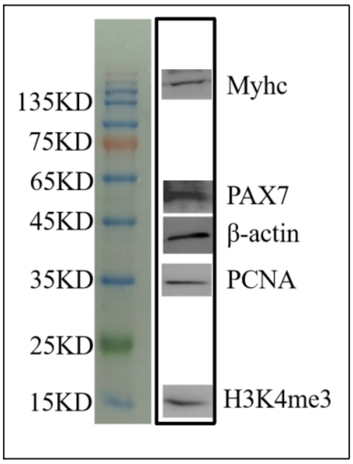

Figure S1. Western blotting (WB) analysis of Myhc, PAX7, PCNA and H3K4me3 expression within Yak skeletal muscle. β-actin was utilized as an internal control. n = 3. (**a**)Cut the whole membrane for blotting of different antibodies, and blots of molecular markers are displayed in each blot. (**b**)Shows the whole blot after cutting membrane.Myhc (220KDa), PAX7(55KDa), PCNA(34KDa) , H3K4me3(15KDa), β-actin(42KDa).
